# Modeling axonal regeneration by changing cytoskeletal dynamics in stem cell-derived motor nerve organoids

**DOI:** 10.1038/s41598-022-05645-6

**Published:** 2022-02-08

**Authors:** Woo Min Seo, Jiyoung Yoon, Ju-Hyun Lee, Yunjeong Lee, Hojae Lee, Dongho Geum, Woong Sun, Mi-Ryoung Song

**Affiliations:** 1 School of Life Sciences, Gwangju Institute of Science and Technology, Oryong-dong, Buk-gu, Gwangju, 61005 Republic of Korea; 2 Department of Anatomy, Brain Korea 21 Plus Program for Biomedical Science, Korea University College of Medicine, 73 Inchon-ro, Seongbuk-gu, 02841 Seoul, Republic of Korea; 3 Cedars-Sinai Medical Center, Biomanufacturing Center, Los Angeles, CA 90069 USA; 4 Department of Biomedical Sciences, Korea University College of Medicine, 73 Inchon-ro, Seongbuk-gu, 02841 Seoul, Republic of Korea

**Keywords:** Neuroscience, Stem cells

## Abstract

Oxidative stress triggers axon degeneration and cell death, leading to the development of neurodegenerative diseases. Spinal motor nerves project very long axons, increasing the burden on axonal transport and metabolism. As such, spinal motor nerves are expected to be susceptible to oxidative stress, but model systems for visualizing and investigating acutely degenerating motor axons are limited. In this study, we establish motor nerve organoids from human pluripotent stem cells (hPSCs) with properties similar to those of neuromesodermal progenitors (NMPs), a population of progenitor cells that comprise the caudal spinal cord. Three-dimensional differentiation of organoids efficiently gave rise to mature motor neurons within 18 days. Adherent organoids showed robust axon fascicles and active growth cones under normal conditions. In addition, more homogenous and efficient generation of motor neurons were achieved when organoids were dissociated into individual cells. Hydrogen peroxide-induced oxidative stress resulted in a broad range of signs of axon degeneration including the disappearance of growth cones and neurites, axon retraction, axon fragmentation and bleb formation, and apoptotic cell death, whose severity can be reliably quantifiable in our culture system. Remarkably, cytoskeletal drugs modulating actin or microtubule turnover differentially facilitated axon dynamics and increased axon regenerative potential. Taken together, our motor nerve organoid model could be potentially useful for drug screens evaluating the rearrangement of cytoskeletons in regenerating motor axons.

## Introduction

Spinal motor nerves extend their long axons to the periphery and innervate muscles for motion control. Motor neurons fire continuously to enable muscles to maintain body posture and perform skilled movements. Unlike other CNS neurons, motor neurons have a large cell soma, very long axons (> 1 m in an adult human), and a large complex structure of neuromuscular junctions. Such unique properties of motor neurons result in massive demand for metabolic energy and axonal transport, rendering this population particularly vulnerable to various types of injuries and diseases. For instance, in both animal models and patients with motor neuron diseases such as amyotrophic lateral sclerosis (ALS), there is selective degeneration of motor neurons, leading to signs of axonal degeneration, mitochondrial dysfunction, cytoskeletal disruption, and muscle denervation. Although the mechanisms underlying the development of ALS are still unclear, numerous studies have shown that oxidative stress in axons could be responsible for progressive development of motor neuron diseases. First, mutations in the *superoxide dismutase 1 (SOD1)* gene, which encodes an anti-oxidant enzyme, are found in about 20% of patients with familial ALS^1^. Second, elevated oxidative damage to protein and DNA is found in tissues from animal models or patients with ALS^2,3^. Third, mitochondrial dysfunction and defective axonal transport are reported in the early stages of ALS disease development, implying that the reactive oxygen species and apoptotic signaling cascade in the axonal compartment of motor neurons could be crucial in disease pathogenesis^4,5^. Thus, investigating the dynamic process of motor axon degeneration in response to oxidative stress is necessary for understanding the basic mechanisms underlying neurodegeneration.

Stem cell-derived motor neurons have provided a promising alternative for studying human motor neurons in pathological conditions. Although a relatively homogeneous motor neuron population can be efficiently derived in dissociated cultures, it may not recapitulate the multicellular organization and microenvironment that exists in vivo. For instance, motor neurons projecting to the same muscle have their cell bodies clustered into discrete nuclei within the neural tube. These so-called motor pools send their axons together to the periphery, forming a tight axon fascicle. To circumvent this problem, we have taken advantage of recent progress in stem cell culture technology to develop three-dimensional (3D) neural organoids, mimicking various regions and cell types within the human brain. Nevertheless, only a few studies have focused on axon projection and connectivity of spinal motor nerves in organoids^6–8^. In this study, we have generated dissociated motor neurons and motor nerve organoids from human pluripotent stem cells (hPSCs) and tested whether they can be useful for monitoring the behavior of axons under various conditions. Motor axon growth on 2D planar surface revealed the entire morphology of individual neurons and apoptotic cell bodies, while growing motor axons were better visualized in 3D organoids showing dynamic growth cones and directional axon growth. When the motor neurons were exposed to conditions of oxidative stress, the axonal compartments underwent a series of pathological changes, such as the disappearance of growth cones, the presence of fragmented axons, and apoptotic cell death. Notably, stabilization of actin and microtubule dynamics led to greater regenerative potential of the axons after nerve injury. We suggest that our motor nerve organoid model recapitulates degeneration and regeneration sequences in vivo and could provide better insights into motor nerve degeneration in humans.

## Results

### Generation of motor nerve organoids

**Figure 1:**
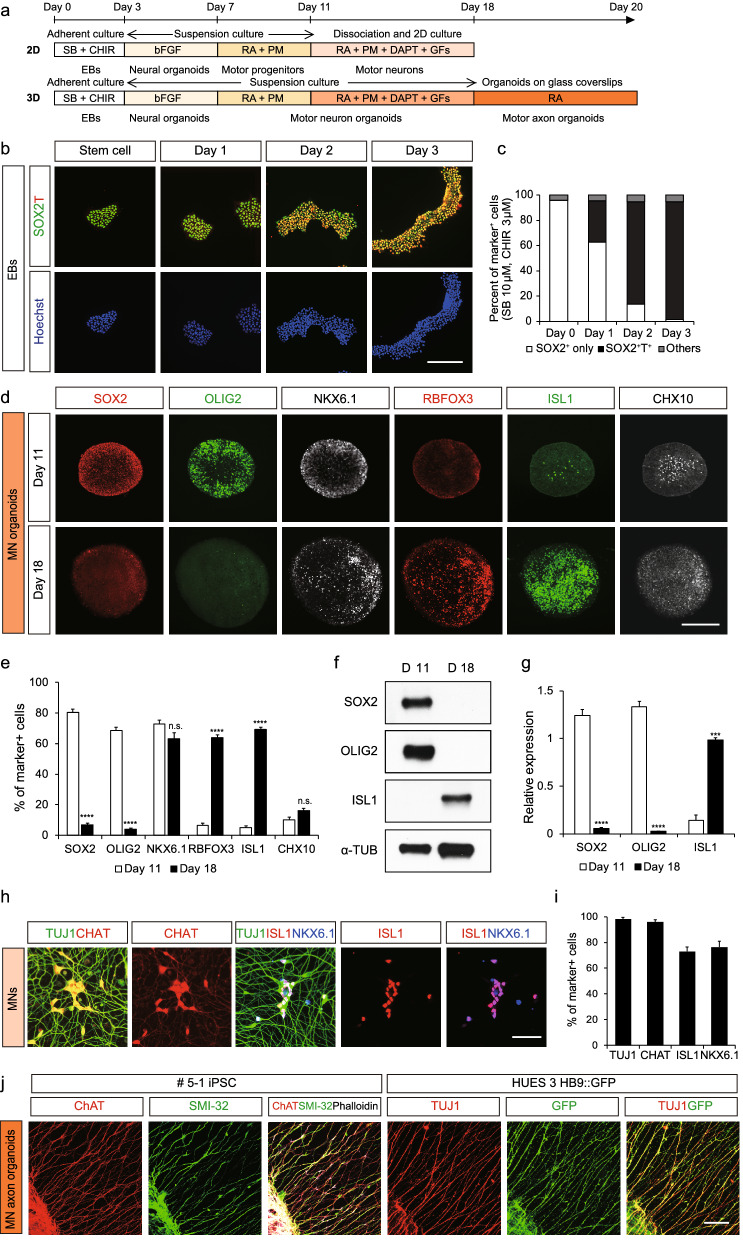
Generation and characterization of motor nerve organoids. (**a**) Schematic of the protocol used to generate dissociated motor neurons and motor nerve organoids. (**b**,**c**) Representatives and quantification of SOX2^+^, T^+^, and SOX2^+^T^+^ cells in organoids attached on the 2D planar surface (n > 9 images per day). (**d**) Images of D11 and D18 organoids immunostained with SOX2, OLIG2, NKX6.1, RBFOX3, ISL1, and CHX10 antibodies. (**e**) Quantification of the percentage of marker-expressing cells among the total number of cells stained with Hoechst dye (n > 9 organoids per group). (**f**) Western blot analysis showing the expression of SOX2, OLIG2, ISL1, and α-tubulin in motor nerve organoids. α-tubulin was used as a loading control. (**g**) Quantification of western blot band intensities (n = 3 experiments). (**h**,**i**) Images and quantification of D18 dissociated motor neurons with neuronal marker TUJ1, motor axonal marker CHAT, and motor neuronal markers ISL1 and NKX6.1 immunoreactivity (n > 3 images per condition). (**j**) Representative images of axons of D18 organoids derived from 5-1 iPSC and HUES 03 HB9::GFP ESCs. Organoids were labeled with motor axon markers CHAT and SMI-32, axonal marker TUJ1, GFP or phalloidin for F-actin as indicated (n = 6 organoids per group). Error bars represent s.e.m.; ***p < 0.001; ****p < 0.0001; n.s., not significant versus Day 11; n.s., not significant; unpaired Student’s t-test. Scale bars 20 μm in (**b**), 200 μm in (**d**), 50 μm in (**h**), 100 μm (left panel) and 250 μm (right panel) in (**j**).

Spinal cord neurons originate from neuromesodermal progenitors (NMPs), a population of progenitor cells that consist of both paraxial mesoderm and caudal neural tube cells^9,10^. We followed a previously published protocol to induce NMP-like cells from human induced pluripotent stem cells (hiPSCs) by applying an inhibitor of TGF-β receptor SB-431542 (10 μM) and a GSK-3 inhibitor CHIR-99021 (3 μM) for 3 days (Fig. 1a)^11^. The NMP markers SOX2 and T were found to be co-expressed in more than 80% of the cells, indicating that most generated cells had acquired NMP identity (Fig. 1b,c). The NMP-like cells then were grown in suspension culture with bFGF for 4 days to form organoids. Subsequent exposure to 0.1 μM RA and the SHH agonist purmorphamine (PM, 1 μM) resulted in ventralization of the organoids. On day 11, more than 80% of cells expressed the progenitor marker SOX2 or markers of ventral progenitor cells such as OLIG2 (68%) and NKX6.1 (73%) (Fig. 1d,e). The low expression of RBFOX3 (NeuN, pan-neuronal marker), ISL1 (motor neuron marker), CHX10 (V2 interneuron marker) on day 11 indicated that the organoids had undergone minimal neuronal differentiation. Application of the notch inhibitor DAPT (10 μM) and growth factors including glial cell-derived neurotrophic factor (GDNF), brain-derived neurotrophic factor (BDNF), ciliary neurotrophic factor (CNTF) and insulin like growth factor-1 (IGF-1) resulted in the efficient conversion of ventral progenitors into mature neurons. On day 18, when most progenitors had differentiated into neurons, the number of progenitors labeled with SOX2 or OLIG2 was found to be reduced (Fig. 1e). Instead, the majority of neurons expressed NeuN (62%), ISL1 (70%), or CHX10 (16%). The proportion of cells expressing NKX6.1 was still high (62%). Of note, NKX6.1 expression was maintained in both ventral progenitors and neurons in vivo. Thus, in our culture condition, organoids consisted mainly of motor neurons (> 70%) and V2 interneurons (16%). Similar changes were observed at the protein level for SOX2, OLIG2, and ISL1 proteins as assessed by western blot analysis (Fig. 1f,g, Fig. S1). Alternatively, we dissociated the spheroids containing motor neuron progenitors at day 11 and grew them in 2-dimensional (2D) culture to evenly expose the cells to DAPT and growth factors including GDNF, BDNF, CNTF and IGF-1 which may produce homogeneous motor neuron population more efficiently. As a result, 98% of cells were differentiated into neurons expressing TUJ1, a marker for immature neurons. Remarkably, most of them also expressed markers related to differentiating motor neurons including CHAT (96%), ISL1 (73%) and NKX6.1 (76%). Similar ratio of cells expressing ISL1 between the 2D and 3D cultures indicates that equivalent level of motor neuron differentiation is achieved in these cultures. Nevertheless, orientation and direction of axonal growth in 2D culture were rather random and dispersed, which barely mimicked the directional and organized growth of axon fascicles composed of multiple axon bundles in vivo. To develop a system to allow afferent axon growth from motor neuronal somata, we attached the organoids onto glass coverslips coated with 100 μg/ml poly-d-lysine (PDL) and 100 μg/ml laminin on a 2D substrate. Most phalloidin-labeled processes expressed CHAT and SMI-32 motor axonal markers, indicating that they were derived from cholinergic mature motor neurons (Fig. 1h,i). We also produced motor nerve organoids from HUES 3 HB9::GFP, an embryonic stem cell (ESC) line carrying a motor neuron–specific GFP reporter under the control of the *HB9* promoter (Fig. 1j)^12^. More than 80% of the TUJ1-expressing axons were found to be labeled with GFP. We therefore successfully established a model system that recapitulates human motor nerve development in vitro.

### Degeneration of motor nerves induced by oxidative stress

**Figure 2:**
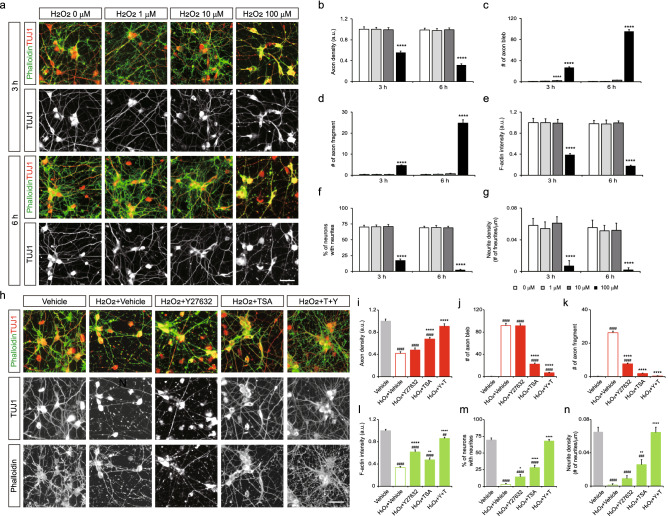
Oxidative stress-driven motor neuronal death and axon degeneration. (**a**) Images of dissociated motor neurons exposed to 1–100 μM of H_2_O_2_ for 3 and 6 h, stained with phalloidin and immunostained with TUJ1 antibody. (**b**–**g**) Measurement of axon density, the number of axonal blebs and fragments, F-actin fluorescence intensity, % of neurons with neurites, neurite density per field (n = 24–36 images per condition). Error bars represent s.e.m.; ****p < 0.0001 versus 0 μM at 3 h or 6 h; one-way ANOVA test with Tukey’s test. (**h**) Images of motor neurons treated with 100 μM of H_2_O_2_ and drugs for 6 h. (**i**–**n**) Quantification of axon density, the number of axonal blebs and fragments, F-actin fluorescence intensity, % of neurons with neurites, neurite density per field (n = 27 images per condition). Error bars represent s.e.m.; ^##^p < 0.01, ^###^p < 0.001, ^####^p < 0.0001 versus Vehicle; *p < 0.05, **p < 0.01, ****p < 0.0001 versus H_2_O_2_ + Vehicle, one-way ANOVA test with Tukey’s test. Scale bars 10 μm.

To test whether the motor neurons we derived can mimic the behavior of axons in vivo upon injury, we exposed neurons to hydrogen peroxide (H_2_O_2_) to induce oxidative damage. At 18 DIV, motor neurons in dissociated 2D culture developed long axons with growth cones at the tip. Motor neurons were then exposed to 1, 10 and 100 μM of H_2_O_2_ for 3 and 6 h (Fig. 2a). Condensed or fragmented nuclei revealed by Hoechst significantly increased in a dose dependent manner (Fig. S2). Signs of axon degeneration were obvious at 100 μM of H_2_O_2_-treated groups in which TUJ1-labeled axons were damaged with lesser axonal density within 3 h (Fig. 2b). Lower concentration of hydrogen peroxide did not cause significant changes in axonal density. A substantial number of axon bleb and fragmented axons appeared in a group with 100 μM of H_2_O_2_ (Fig. 2c,d). Fluorescent intensity of phalloidin-labeled actin structures such as fine neurites and growth cones were significantly dropped relative to the vehicle-treated group (Fig. 2e). The proportion of neurons with filopodia and filopodia density were also dramatically reduced in this condition (Fig. 2f,g). Together, acute treatment of H_2_O_2_ successfully resulted in motor neuronal cell death and axon degeneration and thus we decided to apply 100 μM of H_2_O_2_ to the cells to reliably trigger axon degeneration.

As axon degeneration is accompanied by cytoskeletal disorganization, the regenerative capacity of axons could be enhanced by regulating cytoskeletal dynamics. Since axonal structures are mainly composed of actin filaments and microtubules, we applied the drugs controlling their turnover and measured their potency to sustain axon regeneration. Among various drug candidates commercially available, we chose drugs that meet the following criteria; drugs that have been widely used in pre-clinical studies and proven to be effective in stabilizing actin and tubulin in various physiological and pathological conditions, especially in the context of motor neuron diseases^13–15^, and drugs with well-defined mechanisms whose target molecules are well-characterized in neurons during axon growth or regeneration. Dissociated motor neurons at 18 DIV were treated with 100 μM H_2_O_2_ together with the following drugs for 6 h: the ROCK inhibitor Y-27632 (10 μM) and pan-HDAC inhibitor trichostatin A (TSA, 10 μM), targeting actin filaments and microtubule dynamics, respectively (Fig. 2h)^14^. In the group with H_2_O_2_ treatment, most axons were affected having uneven or discontinuous segments, and obscure and deteriorated growth cones. When the cells were treated with H_2_O_2_ and Y-27632, the extent of neuronal damage did not change, showing similar level of axon density and bleb formation to the group with H_2_O_2_ but axon fragmentation was improved upon Y-27632 (Fig. 2h–k). Actin structures were relatively well-spared determined by increased F-actin intensity (1.7-fold), more number of neurons with neurites (4.6-fold) and greater neurite density (6.5-fold) relative to the group with H_2_O_2_ (Fig. 2h,l–n). Remarkably, when TSA is applied, most axons maintained intact long axons showing 1.6-fold increase in axon density and fewer axon blebbing and fragmentation (Fig. 2h–k). Development of filopodia along the axons was also enhanced comparable to the group with H_2_O_2_ showing a greater F-actin intensity upto 1.5-fold, proportion of neurons with neurites upto 9.1-fold, and density of axonal neurites upto 18.5-fold. Hence, stabilizing microtubules rather than actin filaments was more potent in saving both axons and growth cones from the oxidative damage. Since both drugs regulate two different cytoskeletons and appeared to spare different parts of axonal structures against the oxidative stress, we next tested whether co-treatment of both drugs led to even greater recovery of damaged neurons. Appearance of axons became more close to normal, showing the greatest axon density upto 2.2-fold, F-actin intensity upto 2.6-fold, proportion of neurons with neurites upto 22.1-fold, and filopodia density upto 46.5-fold._._ Therefore, simultaneously stabilizing both cytoskeletons was most effective in saving motor neurons from oxidative stress.

**Figure 3:**
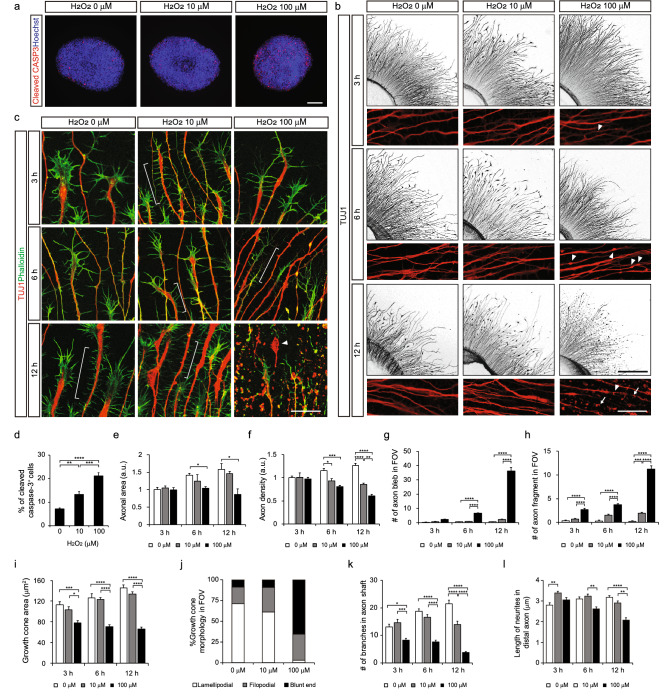
Oxidative stress–induced axon degeneration in motor nerve organoids. (**a**) Representative immunofluorescence images and quantification of organoids treated with H_2_O_2_ for 6 h, stained with apoptosis marker cleaved-CASP3 antibody and Hoechst. Scale bar 100 μm. (**b**) Motor nerve organoids exposed to 10 and 100 μM of H_2_O_2_ for 3, 6, and 12 h, and immunostained with TUJ1 antibody. Arrowheads indicate axon blebs and arrows indicate severed axon fragments. Scale bars 250 μm (upper panels) and 50 μm (lower panels). (**c**) Representative images of growth cones under the oxidative stress in motor axon model, immunostained with TUJ1 antibody and phalloidin. Note that retraction bulb-like deteriorating axon structure (arrowhead) and dramatic reduction of neurites along the axon shaft (compare brackets) in 100 μM of H_2_O_2_–treated group. Scale bar 50 μm. (**d**) Quantification of the percentage of cleaved CASP3^+^ cells among the total number of cells stained with Hoechst dye (n = 9–27 images per condition). (**e**–**h**) Quantification of axonal area per organoid (**e**) (n = 3–16 organoids per condition), axon density per field (**f**) (n = 29–61 images per condition), the number of axon blebs (**g**) (n = 24–36 images per condition), and the number of fragments per field (**h**) (n = 19–33 images per condition). (**i**) Measurement of growth cone area at various time points. (n > 120 growth cones per condition). (**j**) The growth cones at 6 hours were categorized into three types: lamellipodial, filopodial, and blunt growth cones. (n = 91–120 growth cones per group). (**k**, **l**) The number and average length of neurites in axon shafts (< 100 μm from the axon tip) were reduced by H_2_O_2_ treatment (n = 12 axons per condition in **k**, and n = 44–257 neurites per condition in **l**). Error bars represent s.e.m.; *p < 0.05, **p < 0.01, ***p < 0.001, ****p < 0.0001; one-way ANOVA test with Tukey’s test.

Although dissociated motor neurons showed a recovery of damaged axons upon drug treatment, it is unclear whether these drugs were equally effective in motor fascicles in vivo. To test this, D18 organoids were attached to the glass coverslip, which developed actively growing axons with dynamic growth cones within 24 h. Individual axons formed afferent axon projections similar to the axon bundles in vivo. Organoids were then treated with 10 to 100 μM of H_2_O_2_ for 6 h. The number of dying cells detected by cleaved-caspase 3 expression was found to increase at 10 and 100 μM H_2_O_2_, in a dose dependent manner (Fig. 3a,d). Axonal area and density were reduced, and TUJ1-labeled axons developed signs of axon degeneration including axon blebbing and fragmentation within 6 h at 100 μM of H_2_O_2_ (Fig. 3b,e–h). At the lowest concentration, 10 μM H_2_O_2_, axon blebs and fragments were observed to a lesser extent. Next, growth cone structure was visualized by staining F-actin with phalloidin and immunostaining microtubules with anti-TUJ1 antibody. The control group of axons displayed typical morphology of active growth cones with many filopodia and lamellipodia (Fig. 3c). In contrast, growth cones collapsed at 100 μM of H_2_O_2_ within 3 h, showing a reduced area of growth cones, fewer lamellipodial and filopodial structures, and retraction bulb-like swelling at the axon tips (Fig. 3i, j)^16^. After 6 h, axons were found to have completely degenerated, with many severed axon fragments and blebs. The number and average length of neurites on the shaft of distal axons close to the growth cone were dramatically reduced within 3–6 h of H_2_O_2_ treatment (Fig. 3c,k,l). Taken together, our results suggest that motor nerve organoids show multiple signs of cell death and axon degeneration, similar to dissociated motor neurons, when exposed to oxidative stress.

### Motor nerve organoids overcome axon damage by pharmacological modulation of cytoskeletons

**Figure 4:**
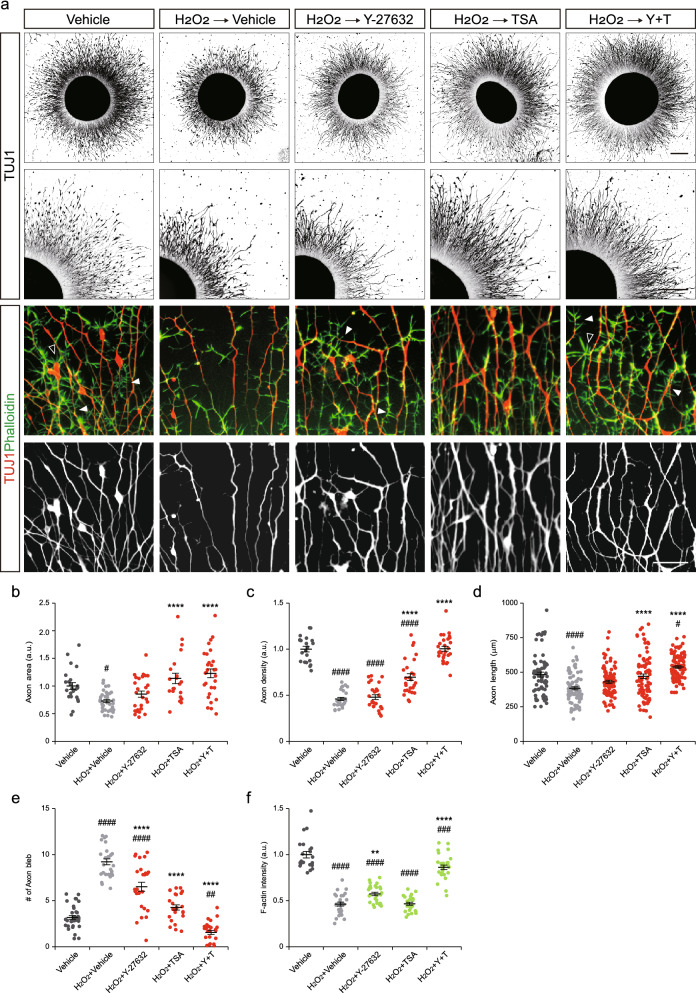
Pharmacological intervention of cytoskeletal dynamics after oxidative stress suppresses axon degeneration. (**a**) Representative images of motor nerve organoids treated with Y-27632 (10 μM) and TSA (10 μM) for 12 h after 100 μM of H_2_O_2_ for 6 h. Lower-panel images show high-magnification views of the upper-panel images. Some fine protrusions at the axon shaft (closed arrowheads) and radially projected filopodial structures (open arrowheads) were marked. Scale bars 250 μm (upper and middle panels), and 30 μm (lower panels). (**b**–**f**) Axonal area (**b**) (n = 24–38 organoids per condition), density (**c**) (n = 21–28 images per condition), length (**d**) (n = 70–90 axons per condition), the number of axon blebs (**e**) (n = 26–37 images per condition), actin fluorescence intensity (**f**) (n = 22–30 images per condition) in drug-treated organoids. Error bars represent s.e.m.; ^#^p < 0.05, ^##^p < 0.01, ^###^p < 0.001, ^####^p < 0.0001 versus Vehicle; **p < 0.01, ****p < 0.0001 versus H_2_O_2_ + Vehicle, one-way ANOVA test with Tukey’s test.

Next, we treated D18 motor nerve organoids that were exposed to oxidative damage with drugs. Since 12 h treatment of hydrogen peroxide almost irreversibly destroyed axons, we chose to apply 100 μM of H_2_O_2_ for 6 h. H_2_O_2_ was added to the culture for 6 h, and then a vehicle, Y-27632 or TSA at a concentration of 10 μM each was added for another 12 h (Fig. 4a). In the control group with no H_2_O_2_ treatment, motor neurons radially extended axons from the organoid. Upon hydrogen peroxide treatment, most axons and growth cones were shortened and collapsed. When Y-27632 was applied with hydrogen peroxide, organoids showed minimal to moderate recovery of damaged axons showing similar level of axonal area, density and length, as well as lesser axon bleb relative to the group with H_2_O_2_ alone (Fig. 4a–e). Thus, stabilizing actin polymerization by lowering the RhoA pathway activity alone partly promotes axon regeneration. In contrast, organoids treated with TSA showed a robust regenerative capacity of axons. Axonal area and density were significantly increased by 1.6-fold and 1.5-fold, and the number of axon blebs were reduced by 2.2-fold. However, fine neurites at the axon shaft with actin filaments were unchanged compared to the group with H_2_O_2_ (Fig. 4f). We next applied both drugs together after H_2_O_2_ treatment. Remarkably, all parameters including the axon area, density and length were greatly improved by 1.4–2.2 fold and the number of blebs decreased by 6.0-fold, when compared to the groups treated with either drug.

**Figure 5:**
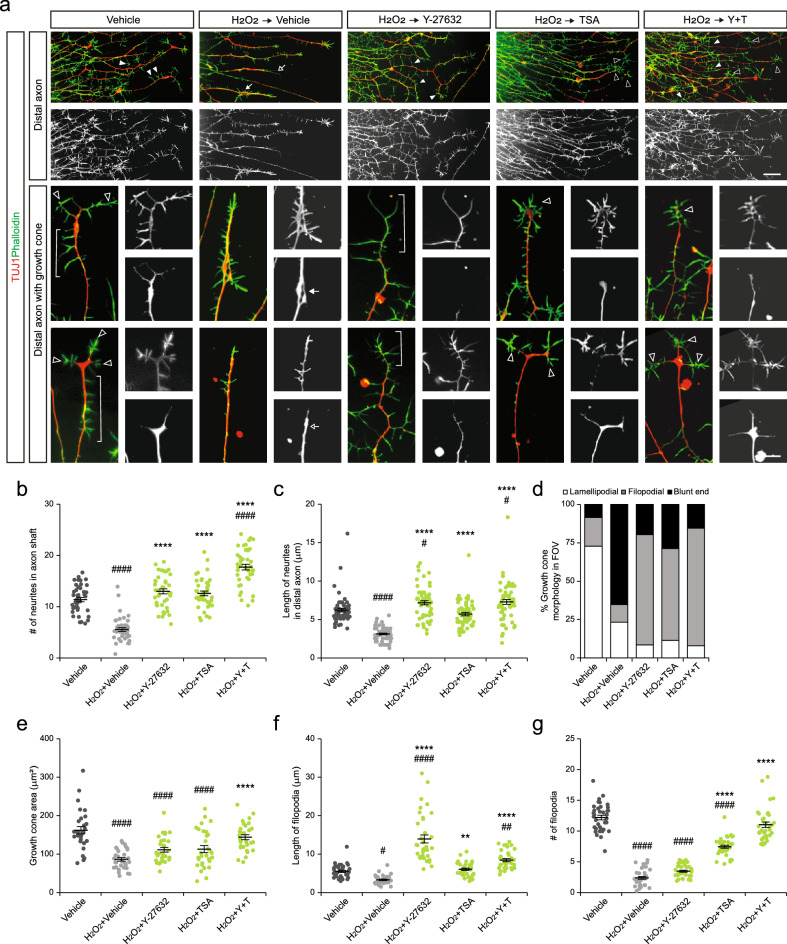
Assessment of regenerating growth cones after cytoskeletal drug treatment. (**a**) Representative images of distal axons and growth cones at the axon tips treated with cytoskeletal drugs. Note short neurites at the axon shaft (closed arrowheads) and diverse morphology of growth cones developing radially projecting filopodia (open arrowheads), elongated filopodia (brackets), disorganized and entangled microtubule network (arrow), or collapsed growth cone (open arrow). (**b**,**c**) The number and length of neurites at the axon shaft (n = 42 axons per condition in b, and n = 60 neurites per condition in **c**). (**d**) Bar graph illustrating the ratio of growth cones with different morphology (n = 61–78 growth cones per condition). (**e**–**g**) The area of the growth cone (**e**) and the number (**f**) and average length (**g**) of neurites in distal axons (< 100 μm from the axon tip) (n = 30 growth cones per condition in **e**, n = 36 neurites per condition in **f** and **g**). Error bars represent s.e.m.; ^#^p < 0.05, ^##^p < 0.01, ^####^p < 0.0001 versus Vehicle; **p < 0.01, ****p < 0.0001 versus H_2_O_2_ + Vehicle, one-way ANOVA test with Tukey’s test. Scale bars 20 μm (upper panels), 5 μm (lower panels).

To determine whether axon extension was associated with dynamic behaviors of axonal tips, we next examined distal axon shaft and growth cones more in detail. We noticed that many short fine neurites developed in distal axon shaft of the control neurons, while they were vanished in neurons exposed to H_2_O_2_ (Fig. 5a–c). When either Y-27632 or TSA were applied after the oxidative stress, more (2.3-fold) and longer (< 1.8-fold) neurites along the shaft of distal axons were found. Remarkably, co-treatment of Y-27632 and TSA resulted in appearance of even greater number of neurites upto 3.2-fold. Next, we examined the morphology of growth cones and categorized them into three groups: filopodial, lamellipodial and blunt end (collapsed with no visible filopodia) (Fig. 5d). In control group, growth cones developed the typical morphology of many actin-rich filopodia in the periphery surrounding the central part of the growth cones with microtubules (Fig. 5a). After H_2_O_2_ exposure, about 65% of growth cones were collapsed. In Y-27632 or TSA-treated group, only 20–29% of growth cones were blunt ended. Thus, both drugs could be effective in reviving the growth cones. However, detailed quantitative analysis of growth cone morphology revealed that these drugs restore different parts of the growth cones. Growth cones in Y-27632-treated group were slightly larger and developed many elongated filopodium, as demonstrated by a greater growth cone area, and the number and length of filopodia (Fig. 5a,e–g). In addition, the central region of the growth cone with microtubules were slender. Growth cones in TSA-treated group were also enlarged and developed more number of filopodia. Of note, the morphology of growth cones in TSA-treated group resembled the normal morphology of actively-growing growth cones with radially projecting filopodium surrounding the slightly enlarged central region of growth cones with microtubules. Thus, Y-27632 is mainly potent in extending actin-based filopodium of the growth cones, while TSA supports overall structure of growth cones organized with microtubules and actin filaments. Lastly, when both drugs were added, the surface area of the growth cones further increased. The length and number of filopodia also increased close to the values in intact growth cones in vehicle control group. Together, simultaneous and acute intervention of actin and microtubule dynamics with Y-27632 and TSA enabled efficient generation of growth cones and maintenance of axon bundles.

## Discussion

An NMP-like population of cells can be induced from PSCs and used to generate spinal motor neurons^9,10^. Within the neural tube, the contribution of NMPs gradually decreases from the posterior to the anterior spinal cord^10^. Thus, in the present study, we used the previously established protocol for the human spinal cord organoid but applied additional ventralizing signals of RA and purmorphamine to efficiently drive the formation of motor neurons located in the ventral horn of the spinal cord^11^. As a result, the majority of the cells expressed mainly the motor neuronal markers OLIG2, ISL1, and NKX6.1. CHX10^+^ V2 interneurons (> 16%) were also generated; this population of cells is found in the ventral spinal cord next to the region where motor neurons arise in vivo. Given that mis-specified motor neurons often choose the V2 interneuronal identity as an alternative fate, we speculate that most cells successfully acquired the ventral cell identity in our condition^17^. After 18 days of differentiation, the organoids had developed long processes that were mostly motor axons co-expressing CHAT, SMI-32, and HB9::GFP reporter^12^. In addition, more uniform and efficient production of motor neurons (> 96%) was obtained when the organoids were dissociated into single cells and were exposed to the cues for motor neuron differentiation. Here we showed that 2D and 3D culture system can validate different aspects of motor axon growth in a complementary manner. For instance, in dissociated 2D culture, morphology of individual neurons can be better visualized, including cell bodies, axons and dendrites. Apoptotic condensed nuclei were easily detectable and sensitive to different doses of oxidative stimuli and drugs in this condition. Motor neurons can be grown easily at a high purity and on a large scale compared to 3D organoids. Thus, 2D dissociated cells can be more suitable for assessing the entire morphology of individual neurons and biochemical analyses requiring large amount of homogeneous samples. In contrast, 3D axon model with organoids provides more in vivo-like environment for spinal motor nerve. Cell bodies were gathered within the organoids resembling motor columns and they were surrounded by other ventral interneurons similar to the spinal cord. Multiple axons exited the organoids together and extended in one direction similar to motor nerve fascicles in vivo. In line with this, we were able to establish various morphological parameters that reliably represent the severity of axon degeneration in a dose dependent manner. More importantly, rapid and sensitive morphological changes of growth cones and fine protrusions in the distal axon were able to distinguish the two different aspect of regenerative potential mediated by actin filaments and microtubules. Taken together, we have established motor nerve models that represents human spinal motor neurons, covering complementary aspects of motor axon growth. Our motor axon models are optimal for gauging the regenerative potential of axons and growth cones.

Unbalanced production of reactive oxidative species causes deleterious consequences in neurons over time that are associated with aging and many neurodegenerative diseases including ALS^2,3,18^. Here we observed the stepwise progression of motor axon degeneration when motor neurons were exposed to H_2_O_2_. Motor axons responded to H_2_O_2_ acutely; collapsed growth cones and retracted axons devoid of neurites were obvious within 3 h. Interestingly, the distal region of retracting axons was rather enlarged and occupied with microtubules, in contrast to normal growth cones developing compact microtubule bundles surrounded by distinct actin arcs segregated from a broad and dynamic actin network. Although the detailed cytoskeletal arrangement needs to be further defined at the molecular level, the retracting axon tip may contain a disorganized microtubule network and disassembled actin arcs, which resembles the retraction bulb, an enlarged distal structure found in damaged axons in vivo^16^. After 6 h, retracted axons underwent degeneration with reduced density and signs of blebbing and fragmentation. Also, at 6 h, there was an increase in apoptotic cell death. Thus, it is likely that the distal axon area became sensitive to oxidative stress and retracted at an early stage and that this may trigger axon degeneration and cell death. The importance of distal axons in neuronal degeneration has been addressed in several studies. When oxidative stress was applied only to the axonal compartment using a microfluidic chamber, local hydrogen peroxide exposure was sufficient to inhibit axonal transport, leading to axonal degeneration and cell death^18^. Another similar system also demonstrated that muscles are more vulnerable to oxidative stress than the neuronal cell body, delivering retrograde signals to the soma and triggering apoptotic cell death^19^. The results of the present study demonstrate that acute responses of distal motor axons to oxidative stress could be an early indicator for gauging the progression of motor neuron degeneration and cell death.

As a strategy to minimize axon degeneration and enhance axon regeneration, we chose to test two representative cytoskeleton drugs that modulate actin filaments and microtubules. ROCK is a downstream effector of the RhoA pathway that is involved in actin dynamics for axon growth and caspase-mediated cell death^20^. During neural development, the RhoA-ROCK pathway inhibits axonal growth and results in growth cone collapse for axon pathfinding. During apoptosis, however, alternative activator caspase-3 cleaves and activates ROCK1, leading to apoptotic membrane blebbing^21,22^. Thus, inhibiting the ROCK pathway could serve a dual function of promoting both axon growth and cell survival. In line with this, treatment of ROCK inhibitor has been proven to be effective in axon growth and regeneration in multiple cellular contexts^23–25^. In this study, blocking the RhoA pathway mostly regenerated actin-related structures including neurites on the axon shaft and filopodium on the growth cones, while the microtubule-based axons whose overall length and density were barely improved. Thus, stabilizing the actin dynamics was not sufficient to fully restore the axonal outgrowth.

More promising results came from the group treated with TSA, a HDAC inhibitor. To date, eighteen HDACs categorized into four classes has been identified in humans. Among them, several HDAC family members are implicated in motor neuron diseases. Inhibition of HDAC1 and HDAC3 is involved in nuclear localization of FUS, while inhibition of HDAC6 prevented recruitment of cytoplasmic FUS to stress granules^26,27^. HDAC1 or HDAC6 also regulate localization and aggregation of Tau and TDP-43 through similar mechanisms^28,29^. Furthermore, HDAC5 and HDAC6 inhibit acetylation of microtubules which reduces their stability and axonal transport^30,31^. In line with this, HDAC inhibitors are proven to be effective in minimizing axon degeneration and cell death in various cellular and pathological contexts^13,14,31^. However, the acute behavior of motor axons and growth cones in response to the oxidative stress and its recovery by increasing the microtubule stability has not been sufficiently investigated yet. In this study, we demonstrated that TSA is highly potent in reversing the axonal damage and boosting the axonal regeneration and outgrowth. Stabilizing the microtubules, which perhaps in turn secured the actin network, were effective in reconstructing the morphology of actively extending growth cones. Due to rather broad spectrum of TSA specificity, it is difficult to pinpoint which HDACs or target molecules are responsible for axon regeneration. Although more rigorous studies to search for the responsible molecules need to be done, considering the fact that better microtubule integrity was observed within 6–12 h of drug incubation, TSA and relevant HDACs are likely to modify tubulin or microtubule-associated proteins, rather than the process requiring gene transcription such as de-differentiation and reprogramming. Finally, when two cytoskeletal drugs were applied together, overall we observed better recovery of injured axons compared to their effect when administered singly. The effect was either synergistic or additive in some of the measures we established, suggesting that targeting both cytoskeletons could be a strategy to maximize the regenerative potential of the drug under the diseased condition. For the several decades, numerous microtubule-actin crosslinking proteins are identified which play a crucial role in consolidating dynamic growth cones and elongating axons^32,33^. Although the detailed molecular mechanisms and the identity of exact cross-linkers need to be further investigated, we speculate that controlling both the regulators of cytoskeleton dynamics and the cross-linkers for cytoskeletal architecture may further maximize the regeneration capacity of neurons.

A variety of cytoskeletal drugs targeting actin or tubulin with greater selectivity and improved side-effect profile have been developed and their efficacies in neurodegenerative diseases has been tested, either in animal or human studies^13,15,34–39^*.* The ROCK inhibitor fasudil, a drug mainly tested on clinical trials for cardiovascular diseases, showed greater recovery in animal models of neurodegenerative diseases^15,38^. Furthermore, a clinical study with fasudil in human ALS patient was recently reported, which may provide us better clue for the action of drugs in the central nervous system^37^. Treatment of TSA also showed improved survival of motor neurons and neuromuscular function in animal models of motor neuron diseases such as ALS and spinal muscular atrophy (SMA)^34,35,39^. Nevertheless, its broad specificity for multiple HDACs and diverse tissue distribution of individual HDACs should be considered before clinical trial^36^. Recently, diverse repertoire of post-translational modification of tubulin known as ‘tubulin code’ has been implicated in various disorders^40^. Thus, assessment of specific cytoskeletal rearrangement molecules and post-translational modification linked to degenerating motor neurons would provide us better clue to develop more precise drug target for clinical trials in the future.

In conclusion, we successfully developed a motor axon model for mimicking the degeneration process of motor nerves using hPSCs and presented multiple biological parameters that can be used to collectively and effectively evaluate the status of degenerating motor axons. Our model could be useful for patient-specific disease modeling and drug screening for motor neuron disease therapies.

## Methods

### iPSC cell culture and formation of motor nerve organoids

Human iPSCs #5-1 were derived from the epidermal fibroblasts of a healthy donor and were provided by Korea University. The HUES 3 HB9::GFP ESC line was purchased from Harvard University^12^. The motor neuron organoids were produced by using a previously described protocol with slight modifications^11^. hPSCs were cultured on Matrigel-coated plates in mTeSR1 (STEMCELL Technologies). After 2 days, mTeSR1 was replaced by differentiation medium DMEM/F-12, supplemented with B27, N2, nonessential amino acids (NEAA), β-mercaptoethanol (Life Technologies), and penicillin/streptomycin (P/S) (HyClone). For NMP differentiation, iPSCs were grown in differentiation medium with SB-431542 (TOCRIS) and CHIR-99021 (SIGMA) for 3 days. Cells were dissociated with Accutase (STEMCELL Technologies) and grown on differentiation media with basic fibroblast growth factor (bFGF) (R&D Systems) to form embryoid bodies for 4 days. Over the following 4 days, developing neurospheres were cultured in differentiation media with 0.1 μM retinoic acid (RA) and 1 μM purmorphamine (CALBIOCHEM) and then additionally with 10 μM DAPT (Tocris) and growth factors (BDNF, CNTF, GDNF, IGF-1, each at 10 ng/ml) (R&D Systems) for 7 days to generate motor nerve organoids.

### Dissociation of motor nerve organoids

Day 11 organoids were dissociated into single cells using Accutase (STEMCELL technologies) in an incubator at 37 °C and 5% CO_2_ for 10 min. No strainer or trituration was applied. 6.5 × 10^6^ cells were plated onto poly-d-lysine (SIGMA) and laminin (Life Technologies)-coated coverslips and were cultured for additional 7 days in DMEM/F12 (Life Technologies) media supplemented with 2% B27, 1% N2, 1% NEAA, and 1% P/S.

### Axon outgrowth on 2D planar surface using motor nerve organoids

Motor nerve organoids were transferred onto glass coverslips coated with poly-d-lysine (SIGMA) and laminin (Life Technologies) and grown in maturation medium (1:1 DMEM/F12 and neurobasal medium [Life Technologies], B27, N2, NEAA, β-mercaptoethanol, GlutaMAX [Life Technologies], P/S, and 1 μM RA). To induce oxidative stress, cultures were treated with H_2_O_2_ (Duksan) in neurobasal media. For the drug experiment, the culture was treated with 100 μM H_2_O_2_ for 6 h. After cell washes, the medium was replaced with fresh medium containing the drugs at a final concentration of 10 μM TSA (Sigma) or 10 μM Y-27632 (Tocris) each. PBS was used as vehicle control. The organoids were grown for additional 12 h before harvest.

### Immunohistochemistry

The organoids were fixed and processed for immunostaining as is or after cryosectioning. Primary antibodies used for immunostaining were as follows: rabbit anti-SOX2 (Millipore), mouse anti-ISL1 (DSHB), rabbit anti-OLIG2 (IBL), mouse anti-NeuN (Chemicon), goat anti-NKX6.1 (R&D Systems), guinea pig anti-CHX10^17^, rabbit anti-TUJ1 (BioLegend), mouse anti-TUJ1 (Millipore), rabbit cleaved-caspase3 (Cell Signaling Technology), goat anti-T (R&D Systems), rabbit anti-GFP (Abcam), goat anti-CHAT (Chemicon), and mouse anti-SMI-32 (BioLegend). After cell washes, samples were incubated with secondary antibodies and counterstained with Alexa Fluor® 488 phalloidin (Invitrogen) for axon labeling and Hoechst 33342 (Thermo Fisher Scientific) for nuclei staining.

### Western blot analysis

Motor nerve organoids were gently homogenized in RIPA lysis buffer (SIGMA) with proteinase inhibitor cocktails (Calbiochem). The lysates were subjected to protein gel electrophoresis by SDS-PAGE, and the proteins were transferred onto nitrocellulose membrane by electrophoresis. The membranes were trimmed before hybridization and were incubated with primary and secondary antibodies. The blots were developed with the ECL Plus chemiluminescence reagent kit (Thermo Fisher Scientific). The band intensities were measured by ImageJ and normalized to α-tubulin band levels.

### Image analysis

The number of cells was counted in a randomly selected square with the size of 63 × 63 μm^2^ and normalized to the number of Hoechst^+^ cells. To measure the area of axon growth, the area of the neurosphere mass was subtracted from the area of the entire organoid with axons. Axon density was measured by averaging the pixel intensities of four randomly selected squares, each with a size of 128 × 128 μm^2^ for dissociated cells or 100 × 100 μm^2^ for organoids. To count the number of axon blebs, fragments and % of neurons with neurites, > 24 squares were randomly selected in dissociated culture. To measure neurite density, 27 axons were randomly selected and the number of neurites were counted in every 40 μm. For organoids, three or four squares (for axon blebs, 100 × 100 μm^2^; for fragments, 25 × 50 μm^2^) located 100 μm away from the boundary of organoids were randomly selected for quantification. For measurements of neurites in the distal axon, protrusions that were stained with phalloidin and were longer than 1 μm in length and located 100 μm away from the axon tip were analyzed. Growth cones were analyzed from a square with the size of 128 × 128 μm^2^ for dissociated cells or 100 × 100 μm^2^ for organoids. The growth cone area was measured by tracing the boundary of the area labeled with phalloidin in the axon tip and included both the filopodia and lamellipodia area using ImageJ. Growth cones were classified into three types based on morphology: lamellipodial growth cones had a broad well-spread meshwork of actin filaments, filopodial growth cones had long actin-rich protrusions, and blunt growth cones had no protruding filopodia or lamellipodia. For measurement of the number and length of filopodia, the number and length of long fine processes developed from the boundary of the central domain of the growth cone was quantified.

### Statistical analysis

All data are expressed as the mean ± SEM, obtained from at least three independent experiments. For each experiment, data was acquired from 3–4 fields of view per organoid for 5–30 organoids, pooled and analyzed unless noted. Mean values per experiment, were then averaged across experiments. Statistical analysis was performed using GraphPad Prism 9 software. Data were subjected to unpaired-*t*-test or one-way ANOVA with Tukey’s test for multiple comparisons.

## Supplementary Information

Supplementary Information.

## Data Availability

The datasets analyzed during the current study are available from the corresponding author on reasonable request. All methods were carried out in accordance with relevant guidelines and regulations.

## References

[1] Cudkowicz ME (1997). Epidemiology of mutations in superoxide dismutase in amyotrophic lateral sclerosis. Ann. Neurol..

[2] Ferrante RJ (1997). Evidence of increased oxidative damage in both sporadic and familial amyotrophic lateral sclerosis. J. Neurochem..

[3] Shaw PJ, Ince PG, Falkous G, Mantle D (1995). Oxidative damage to protein in sporadic motor neuron disease spinal cord. Ann. Neurol..

[4] De Vos KJ (2007). Familial amyotrophic lateral sclerosis-linked SOD1 mutants perturb fast axonal transport to reduce axonal mitochondria content. Hum. Mol. Genet..

[5] Sasaki S, Iwata M (1996). Impairment of fast axonal transport in the proximal axons of anterior horn neurons in amyotrophic lateral sclerosis. Neurology.

[6] Faustino Martins JM (2020). Self-organizing 3D human trunk neuromuscular organoids. Cell Stem Cell.

[7] Andersen J (2020). Generation of functional human 3D cortico-motor assembloids. Cell.

[8] Kawada J (2017). Generation of a motor nerve organoid with human stem cell-derived neurons. Stem Cell Rep..

[9] Henrique D, Abranches E, Verrier L, Storey KG (2015). Neuromesodermal progenitors and the making of the spinal cord. Development.

[10] Garriock RJ (2015). Lineage tracing of neuromesodermal progenitors reveals novel Wnt-dependent roles in trunk progenitor cell maintenance and differentiation. Development.

[11] Lee J-H, Shaker MR, Kim HJ, Kim JH, Lee N, Kang M, Cho S, Kwak TH, Kim JW, Song M-R, Kwon S-H, Han DW, Lee S, Choi S-Y, Rhyu IJ, Kim H, Geum D, Cho I-J, Sun W (2020). Human spinal cord organoids exhibiting neural tube morphogenesis for a quantifiable drug screening system of neural tube defects. bioRxiv.

[12] Di Giorgio FP, Boulting GL, Bobrowicz S, Eggan KC (2008). Human embryonic stem cell-derived motor neurons are sensitive to the toxic effect of glial cells carrying an ALS-causing mutation. Cell Stem Cell.

[13] Klingl YE, Pakravan D, Van Den Bosch L (2021). Opportunities for histone deacetylase inhibition in amyotrophic lateral sclerosis. Br. J. Pharmacol..

[14] Rivieccio MA (2009). HDAC6 is a target for protection and regeneration following injury in the nervous system. Proc. Natl. Acad. Sci. USA.

[15] Koch JC (2018). ROCK inhibition in models of neurodegeneration and its potential for clinical translation. Pharmacol. Ther..

[16] Riley DA (1981). Ultrastructural evidence for axon retraction during the spontaneous elimination of polyneuronal innervation of the rat soleus muscle. J. Neurocytol..

[17] Thaler J (1999). Active suppression of interneuron programs within developing motor neurons revealed by analysis of homeodomain factor HB9. Neuron.

[18] Fang C, Bourdette D, Banker G (2012). Oxidative stress inhibits axonal transport: implications for neurodegenerative diseases. Mol. Neurodegener..

[19] Zahavi EE (2015). A compartmentalized microfluidic neuromuscular co-culture system reveals spatial aspects of GDNF functions. J. Cell Sci..

[20] Fujita Y, Yamashita T (2014). Axon growth inhibition by RhoA/ROCK in the central nervous system. Front Neurosci..

[21] Coleman ML (2001). Membrane blebbing during apoptosis results from caspase-mediated activation of ROCK I. Nat. Cell Biol..

[22] Sebbagh M (2001). Caspase-3-mediated cleavage of ROCK I induces MLC phosphorylation and apoptotic membrane blebbing. Nat. Cell Biol..

[23] Lingor P (2007). Inhibition of Rho kinase (ROCK) increases neurite outgrowth on chondroitin sulphate proteoglycan in vitro and axonal regeneration in the adult optic nerve in vivo. J. Neurochem..

[24] Fournier AE, Takizawa BT, Strittmatter SM (2003). Rho kinase inhibition enhances axonal regeneration in the injured CNS. J. Neurosci..

[25] Joshi AR, Muke I, Bobylev I, Lehmann HC (2019). ROCK inhibition improves axonal regeneration in a preclinical model of amyotrophic lateral sclerosis. J. Comp. Neurol..

[26] Kuta R (2020). Depending on the stress, histone deacetylase inhibitors act as heat shock protein co-inducers in motor neurons and potentiate arimoclomol, exerting neuroprotection through multiple mechanisms in ALS models. Cell Stress Chaperones.

[27] Kwon S, Zhang Y, Matthias P (2007). The deacetylase HDAC6 is a novel critical component of stress granules involved in the stress response. Genes Dev..

[28] Tseng JH (2017). The deacetylase HDAC6 mediates endogenous neuritic tau pathology. Cell Rep..

[29] Sanna S (2020). HDAC1 inhibition ameliorates TDP-43-induced cell death in vitro and in vivo. Cell Death Dis.

[30] Hubbert C (2002). HDAC6 is a microtubule-associated deacetylase. Nature.

[31] Cho Y, Sloutsky R, Naegle KM, Cavalli V (2013). Injury-induced HDAC5 nuclear export is essential for axon regeneration. Cell.

[32] Coles CH, Bradke F (2015). Coordinating neuronal actin-microtubule dynamics. Curr. Biol..

[33] Rodriguez OC (2003). Conserved microtubule-actin interactions in cell movement and morphogenesis. Nat. Cell Biol..

[34] Yoo YE, Ko CP (2011). Treatment with trichostatin A initiated after disease onset delays disease progression and increases survival in a mouse model of amyotrophic lateral sclerosis. Exp. Neurol..

[35] Avila AM (2007). Trichostatin A increases SMN expression and survival in a mouse model of spinal muscular atrophy. J. Clin. Invest..

[36] Lunke S, El-Osta A (2013). Applicability of histone deacetylase inhibition for the treatment of spinal muscular atrophy. Neurotherapeutics.

[37] Koch JC (2020). Compassionate use of the ROCK inhibitor Fasudil in three patients with amyotrophic lateral sclerosis. Front Neurol..

[38] Takata M (2013). Fasudil, a rho kinase inhibitor, limits motor neuron loss in experimental models of amyotrophic lateral sclerosis. Br. J. Pharmacol..

[39] Rossaert E (2019). Restoration of histone acetylation ameliorates disease and metabolic abnormalities in a FUS mouse model. Acta Neuropathol. Commun..

[40] Janke C (2014). The tubulin code: Molecular components, readout mechanisms, and functions. J. Cell Biol..

