# Supplementary material for: Modeling axonal regeneration by changing cytoskeletal dynamics in stem cell-derived motor nerve organoids
Source: Sci Rep. 2022 Feb 8;12:2082. doi: 10.1038/s41598-022-05645-6 (PMC8827082; doi:10.1038/s41598-022-05645-6)
Supplement: Supplementary file 1 — Supplementary Information. [file 41598_2022_5645_MOESM1_ESM.pdf]

**Supplementary information**

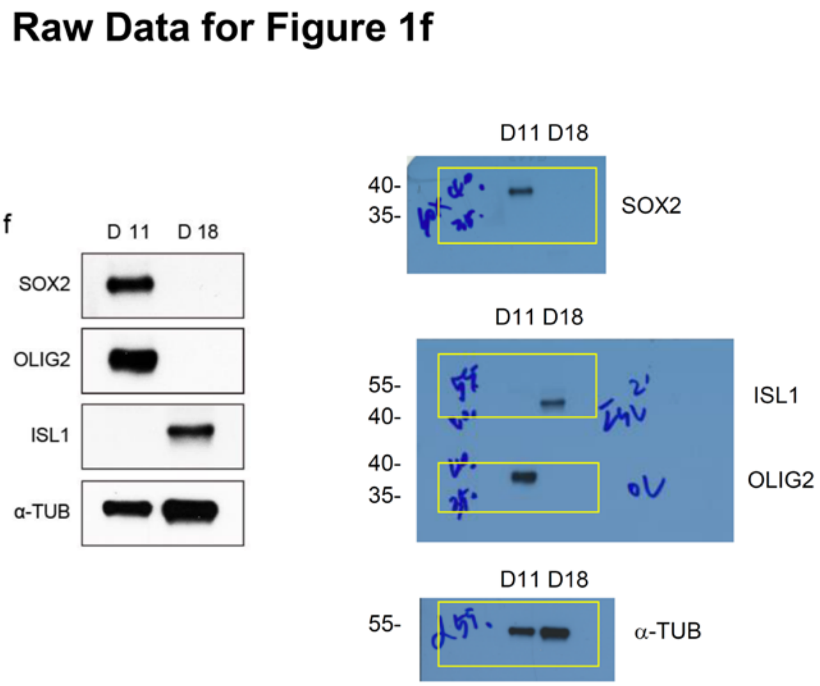


**Sup. Fig. 1.** The original images for blots in Fig. 1f

The blots were trimmed before hybridization with antibodies. Individual trimmed original blots were marked in yellow boxes.

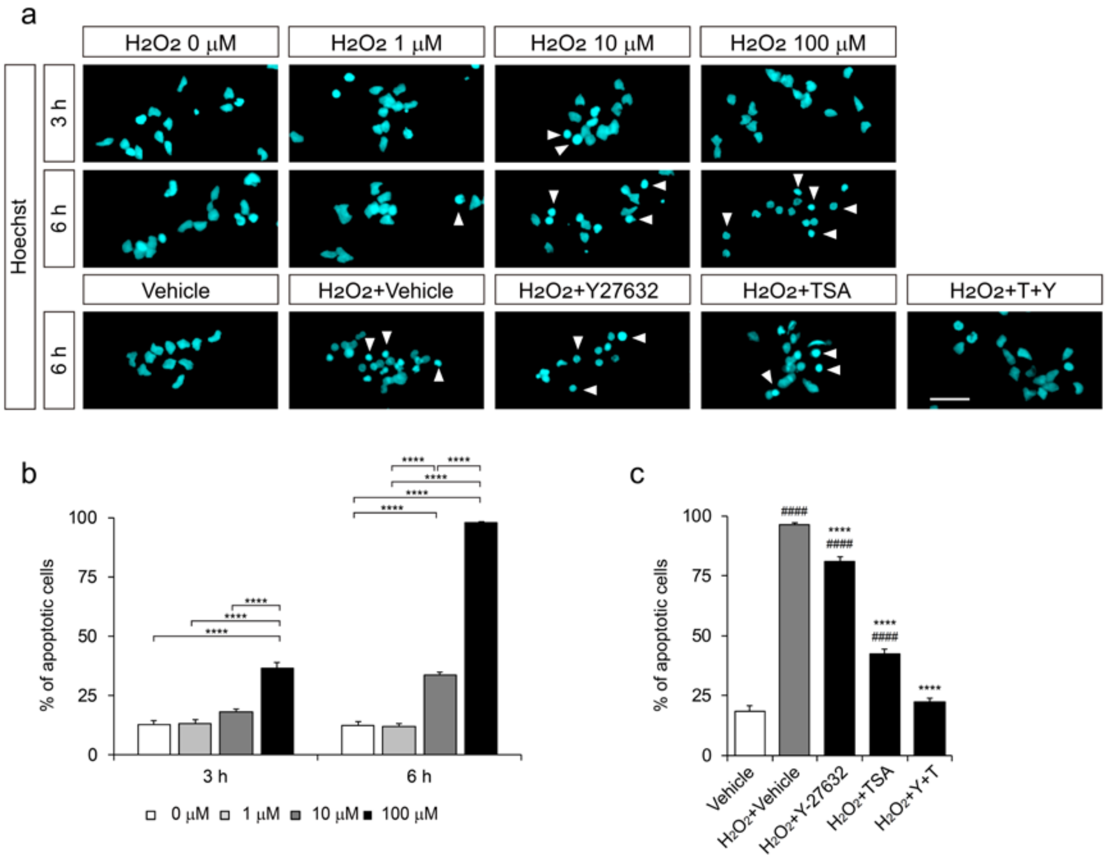


**Sup. Fig. 2.** Assessment of apoptotic nuclei after the oxidative stress

(a) Representative images of Hoechst-stained cell bodies in various conditions of hydrogen peroxide and drug treatment. Arrowheads indicate bright punctate apoptotic nuclei. (b, c) Quantification of apoptotic cell bodies (n = 27 images per condition from 3 independent experiments). Error bars represent s.e.m.; ****$p<0.0001$; one-way ANOVA with Tukey's test (b). Error bars represent s.e.m.; ####$p<0.0001$ versus Vehicle; ****$p<0.0001$ versus $H_2O_2$+Vehicle, one-way ANOVA test with Tukey's test (c). Scale bar: 10 μm.
